# Reliability of a Deep Learning Model in Predicting Permanent Maxillary Canine Eruption for Preventive Orthodontics: A Comparative Study with Orthodontists

**DOI:** 10.3390/diagnostics16142172

**Published:** 2026-07-11

**Authors:** Zana Qadir Omer

**Affiliations:** Dentistry College, Hawler Medical University, Erbil 44001, Iraq; zana.qadir@hmu.edu.krd

**Keywords:** deep learning model, unerupted canine, AI, OPG, preventive orthodontics, Modified Ericson and Kurol sectors

## Abstract

**Background/Objective:** The management and prevention of impacted maxillary canines is both an art and a science. These important teeth serve both aesthetic and functional purposes, so they demand meticulous evaluation. Deep learning model tools are game-changing technologies, elevating diagnostic capabilities and therapeutic planning. This study comprised a comparative analysis of the prediction of permanent maxillary canine eruption as a preventive measure between experts and a deep learning model. **Methods**: In this retrospective cross-sectional study, 2230 panoramic radiographs of patients aged 9–14 years were analyzed to assess the patterns of eruption of unerupted maxillary permanent canines (UPMCs). The study images were classified into three sectors according to the modified Ericson and Kurol sectors. Data preprocessing techniques were used to prepare images for the deep learning model by using a DenseNet121-based Convolutional Neural Network (CNN). The data were split into training and testing sets to train the AI to predict sectors. The deep learning model’s predictions were evaluated using sector accuracy, precision, recall, and F1 score. **Results:** The sample included 796 (35.6%) men and 1434 (64.3%) women. The mean age of the participants was 12.2 ± 1.60 years. For sector 1, the right side of UMPCs, the AI reported an accuracy of 99.55% and perfect precision at 100%, while for the left side, accuracy and precision were 99.24% and 100.00%, respectively. For sector 2, prediction on the right side, the performance accuracy values reached 99.73%, and the precision was 98.15%. For the left side of UMPCs, the prediction accuracy was 98.79%, and precision was 94.10%. Regarding sector 3, the right side saw 99.82% accuracy and 98.83% precision. For the left side, the AI achieved an overall accuracy of 99.46% and precision of 99.72%. **Conclusions**: The deep learning-based system significantly reduced the time and human resources required for landmark identification and parameter generation, making the diagnostic process more efficient in interceptive/preventive orthodontics.

## 1. Introduction

Maxillary canines, often referred to as the “cornerstones” of the dental arch, are second only to third molars in terms of tooth impaction, affecting around 2% of the population. This condition, which is more common in women, often presents in young individuals, with a prevalence of 1–3% [1]. Interestingly, impaction occurs twice as frequently in the maxilla as it does in the mandible, and approximately 8% of cases involve both sides. While some impacted canines are positioned labially, the majority—about two-thirds—are located palatally [2].

The reasons for canine impaction are varied, ranging from genetic predispositions to localized and systemic factors [3,4]. Addressing this issue as early as possible is critical for interceptive treatment. Abnormal eruption of the maxillary canine is possibly linked to root resorption of the central and lateral incisors or extreme bone retention of the canine, which may lead to its surgical disclosure and orthodontic traction in the dental arch, which significantly affects not just the function of the teeth but also overall facial aesthetics and appearance [5].

Interceptive and preventive orthodontic treatment often involves early extraction of the deciduous canine at certain ages; these interventions reduce the risk of malocclusion and the need for more complex orthodontic treatment [6,7].

Although there is no unified protocol for defining the tendency toward canine impaction, the most effective treatment approach depends on the early diagnosis of impacted canines and prevention of potential impaction [8]. One notable finding by Ericson and Kurol is that removing primary canines before the age of 11 can help permanent canines erupt properly in most cases and will change the eruption position of the permanent maxillary canines in 91% of cases when the canine crown position is distal to the axial line of the lateral incisor. However, if the canine crown tip is mesial to the axial line of the lateral incisor, the success rate will decrease to 64% [9]. Proper diagnosis requires careful observation of clinical signs, such as delayed eruption or unusual bulges on the palate of the permanent canine teeth after the age of 14 or 15, alongside radiographic imaging such as panoramic radiographs, periapical images, and occlusal films, which are essential tools in determining the location and nature of the impaction [10].

To aid in diagnosis and treatment planning, classification systems like Ericson and Kurol’s five-sector method and Yamamoto’s seven-subtype system categorize canine impaction based on radiographic features [9,10,11]. These frameworks not only standardize evaluations but also help guide early diagnosis of unerupted canines, which may reduce the treatment time, orthodontic treatment complications, and canine loss [12,13].

Artificial Intelligence (AI), including implementations of Deep Learning (DL) and Machine Learning (ML), has been integrated into many aspects of life, including the medical field. AI is a very general term for imitation: it is the copying of human activities so that a computer can perform them more easily and faster [14].

Specialized algorithms create mathematical models that extrapolate patterns to an outcome of decisions that have not been explicitly written to perform that particular task [15]. Recent advancements in these technologies offer hope for more accurate, efficient, and consistent diagnoses. Convolutional Neural Networks (CNNs), a type of deep learning model, have proven particularly effective in medical imaging tasks, such as tooth segmentation, object detection, classification, and anomaly detection [16,17,18]. Convolutional Neural Networks (CNNs) are a specialized type of neural network designed for processing structured grid data, such as images. They have gained popularity and success in image classification tasks due to their ability to automatically learn and extract features from images, which is crucial for accurate classification [19].

By mimicking the human brain’s ability to learn and identify patterns, CNNs can analyze complex data sets, providing dentists with precise insights and reducing the likelihood of errors [20].

Beyond dentistry, AI has transformed various medical fields by enhancing decision-making, improving diagnostic accuracy, and personalizing treatments. Within orthodontics, the application of AI to impacted canine classification represents a step toward streamlining care. For example, DL systems can rapidly measure angles and distances, taking into account factors that might otherwise be overlooked in manual assessments. This not only saves time but also helps reconcile differing opinions among clinicians by offering objective, data-driven evaluations [21,22].

The management of affected maxillary canines is both an art and a science. These important teeth serve a dual purpose—appearance and function—so they require meticulous attention. The use of AI tools serves as a game-changing technology, elevating diagnostic capabilities and therapeutic planning. The results improve patient outcomes and enhance workflows by combining clinical expertise with modern technology. Despite the potential benefits, automated canine impaction classification remains a relatively unexplored area. To date, few studies have employed DL to classify canine impactions using advanced systems such as Ericson and Kurol or Yamamoto’s classification. The current research aims to fill this gap by developing and validating DL models capable of distinguishing between specific impaction subtypes. Such advancements have the potential to not only improve diagnostic accuracy but also enhance patient outcomes by facilitating earlier and more targeted interventions.

This study aims to compare the performance of a deep learning AI system versus orthodontic experts in identifying the position of unerupted maxillary permanent canines (UMPCs) in mixed dentition and grouping them according to Ericson and Kurol sectors on panoramic radiographic images.

## 2. Materials and Methods

### 2.1. Study Design

This study was designed as a retrospective cross-sectional study based on a review of panoramic radiographs from patients who attended the outpatient clinics at the College of Dentistry, Hawler Medical University, Iraq. Ethical approval was obtained and approved by the Research and Ethical Committee of the Hawler Medical University/College of Dentistry, Erbil, Iraq (reference number HMU245002, 22 December 2024). Informed consent was obtained from the parents or legal guardians of all participants involved in the study.

The study was designed as a diagnostic accuracy study aimed at evaluating and comparing the prediction of the eruption of permanent maxillary canines (UMPCs) as a preventive measure between experts and a deep learning model using panoramic radiographs.

### 2.2. Sample and Dataset Description

A total of 1250 OPG cases with uni- or bilateral unerupted maxillary permanent canines (UMPCs) were collected between 29 December 2024 and 30 April 2025 from the radiology database of the Diagnosis and Radiology Department in the College of Dentistry teaching clinics (Hawler Medical University, Erbil). Cases of bilateral unerupted canines were also included in the total sample and were calculated separately as right and left cases; because the eruption path, angulation, sector position, and severity of impaction of each permanent maxillary canine can vary from right to left within the same patient, each canine was treated as an independent observational unit. The total sample comprised 2230 subjects (796 men and 1434 women). Their ages ranged from 9 to 14 years and they had visited the Dental College Clinics at Hawler Medical University. This age interval represents the normal time for permanent eruption of the maxillary canine tooth and covers the normal biological variation in eruption time among different individuals.

The retrospective data included patients referred for a range of dental, orthodontic, pedodontic, and maxillofacial conditions, with a focus on cases involving suspected impacted canines. To reduce bias, a diverse dataset was utilized. The position of maxillary canines and their eruptive path were radiographically investigated in patients in whom clinical investigation had indicated a disturbance in eruption.

**The inclusion criteria were** (1) chronological age range 9–14 years, (2) all teeth present with/without the third molars, (3) no crown or bridge restoration, and (4) no interproximal caries or restoration.

**Exclusion criteria included** (1) patients with severe dental anomalies in the anterior maxillary sextant and a congenitally missing permanent upper lateral incisor, (2) images with significant artifacts such as motion artifacts or metal streak artifacts, (3) patients who had previous orthodontic treatment, or (4) exfoliated deciduous upper canines.

Panoramic images were obtained for each subject using the Vatech PaX-i3D (Model PHT6500) from Vatech, Hwaseong, Republic of Korea, which features a 0.5 mm focal spot compliant with IEC 60336 standards. It operates at 90 kV and 10 mA with a total filtration of 2.8 mm AI. The machine’s software automatically adjusts the radiation settings and exposure time for OPG imaging based on the patient’s age. The reported measurements were adjusted 1:1 according to the magnification factor. Data regarding patient age and sex were obtained from the patients’ main data. To protect patient privacy, all image files were coded before being made available to the observers. Before the radiographs were analyzed by a deep learning model, all panoramic radiographs were investigated by comparing measurements taken by the researcher on two separate occasions, two weeks apart, to assess the intra-examiner reliability for 106 panoramic radiographs. At the same time, the inter-examiner reliability was determined by comparing measurements obtained by the researcher and two other independent experts on the same 106 samples. The agreement between these examiners was quantified.

**Figure 1:**
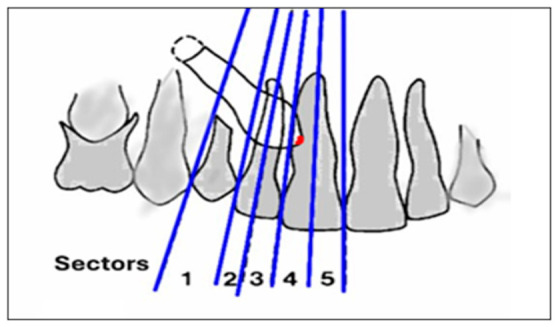
Schematic representation of the Sector method by Ericson and Kurol. 1, 2, 3, 4, and 5: Sector area. Adapted from Ericson S, Kurol J. (1988) [9].

The assessment of unerupted maxillary canines was performed according to Ericson and Kurol [9], as follows (Figure 1):

Sector 1: The cusp tip of the canine is distal to the root of the lateral incisor.

Sector 2: The cusp tip of the canine overlaps the distal half of the root of the lateral incisor.

Sector 3: The cusp tip of the canine overlaps the mesial half of the root of the lateral incisor.

Sector 4: The cusp tip of the canine overlaps the distal half of the root of the central incisor.

Sector 5: The cusp tip of the canine overlaps the mesial half of the root of the central incisor.

**Figure 2:**
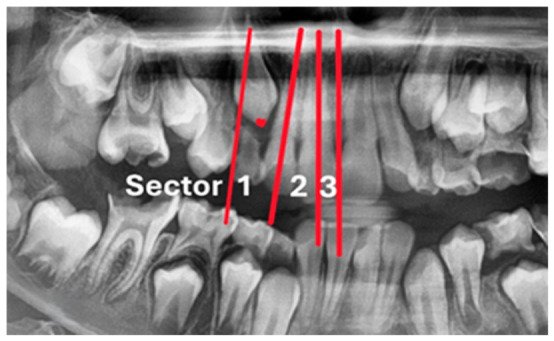
Modified Ericson and Kurol UMPC sector classification.

To facilitate the comparison of the position of the impacted canine and simplify the analysis and the training of AI mechanisms, we modified and consolidated the Ericson and Kurol sectors into three groups (Figure 2):

Sector 1: The area between the line bisecting the contact point of the upper 1st premolar with the deciduous canine and the midline of the upper lateral incisor (Sectors 1 and 2).

Sector 2: The area between the midline of the upper lateral incisor and the midline of the upper central incisor (Sectors 3 and 4).

Sector 3: The area between the midline of the upper central incisor and the line bisecting the contact point of the upper right and left central incisors (Sector 5).

#### Data Preprocessing: (Image Preprocessing), Figure 3

This research employs a deep learning technique, specifically the DenseNet121 model, to automate diagnostic assessments of unerupted permanent maxillary canines. The key feature of this model is its ability to make predictions of UMPCs within the defined area sectors.

**Figure 3:**
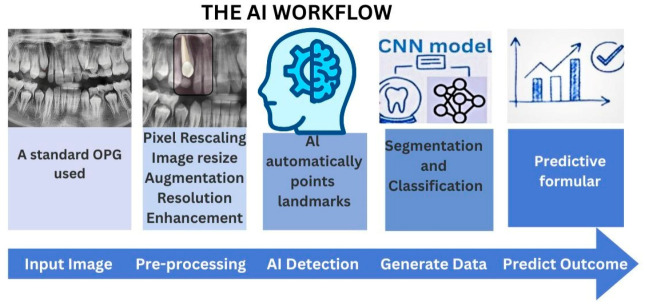
Data processing for a deep learning model.

**Image preprocessing** involves transforming the original data to enhance the model’s learning capabilities before feeding it into the classification model.

The following techniques were used:

**Pixel Rescaling**: Adjusting the pixel values to a specific range to standardize the input data.

**Image Resize**: Resizing images to a uniform dimension to ensure consistency across the dataset, which is essential for deep learning models.

**Augmentation:** Techniques such as image flipping, rotation, and zooming are applied to increase the variability of the dataset. This helps the model generalize better by exposing it to a wider range of examples.

**Resolution Enhancement:** Improving the quality of the images to ensure that the features necessary for classification are more discernible.

These are necessary steps to prepare the data so that it can be effectively used to train and test the convolutional neural network (CNN) architectures that will be used in the study, and ultimately improve the accuracy of UMPC classification [23].

After the data has been loaded, the next step is to split it into two sets: a training set and a testing set [24]. The model learns by looking for patterns and finding any mistakes it makes when making predictions on the training set. This helps in fine-tuning the model’s parameters to improve performance [25].

The testing set is used for a different purpose. It tests the effectiveness of the model by using data that the model has not previously seen and can provide a more accurate overview of the model and the frequency of its errors. This is crucial for ensuring that the model is prepared to handle real-world data. Figure 3 explains the AI workflow and data processing.

In summary, the workflow in this study was designed to be straightforward and clinically practical, as follows:

1. A routine panoramic radiograph is uploaded into the system.
2. An orthodontist examines and classifies the unerupted canines.
3. The artificial intelligence algorithm then automatically identifies and marks all landmarks (removing the need for manual tracing); see Figure 3 (five stages of AI workflow).
4. Finally, these measurements are integrated into a predictive model that generates an output to support the clinician in treatment planning.

This study aims to conduct a comparative analysis UMPC prediction as a preventive orthodontic measure between experts and a deep learning model using panoramic radiographs. This research seeks to explore the potential of artificial intelligence in enhancing diagnostic precision, accuracy, and efficiency.

The null hypothesis (H_0_) is that Sectors 1, 2, and 3 will have the same test scores, while an alternative hypothesis (H_1_) posits a difference.

### 2.3. Statistical Analysis

The reliability of intra- and inter-examiner data readings was evaluated using the Kruskal–Wallis H test, which is a non-parametric alternative to one-way ANOVA suitable for non-normally distributed data. The H statistic, mean rank per group, and *p*-value were reported for each measurement variable. The descriptive statistics for general sample characteristics included frequencies and percentages for categorical variables and the mean, minimum, maximum, and range for continuous variables. Associations between categorical variables were assessed using the Pearson Chi-Square (χ^2^) test. Fisher’s Exact Test was used when expected cell counts fell below five.

We compared the diagnostic performance of the experts and the deep learning model by using a confusion matrix to derive accuracy, precision (positive predictive value), recall (sensitivity), and the F1 Score (harmonic mean of precision and recall). The overall discriminative ability was further measured by the Receiver Operating Characteristic (ROC) curve and the Area Under the Curve (AUC). All analyses were conducted using IBM SPSS (v.27.0) and MedCalc (v.23.4.0), with statistical significance set at *p* < 0.05.

## 3. Results

Panoramic images of 2230 subjects (796 males and 1434 females) were selected for this study. Their ages ranged between 9 and 14 years old. The mean age of the patients was 12.23 ± 1.60 years. The data obtained from all 1250 panoramic radiographs were divided into different age groups; for cases with UMPCs on both sides (right and left), each side was assessed separately. In other words, the right and left canines were counted as two independent image samples. Therefore, the total sample size was calculated according to the number of UMPC sites examined rather than the number of patients; accordingly, the total sample size was 2230.

To demonstrate the reliability of our data, inter- and intra-examiner agreement was evaluated. The intra-examiner reliability was assessed by comparing measurements taken by the researcher on two separate occasions, two weeks apart, for 106 panoramic radiographs. The consistency of these repeated measurements was evaluated using the Mann–Whitney test, which showed that the differences between the results of Intra 1 vs. Intra 2 were not significant. This indicates a high degree of consistency in measurements performed by the same examiner over time (as shown in Table 1).

Inter-examiner reliability was determined by comparing measurements obtained by the researcher and another two independent examiners on the same 106 samples. The agreement between these examiners was quantified using the Kruskal–Wallis test. The results demonstrated excellent inter-examiner reliability, with a *p*-value of 0.614. This finding suggests strong agreement between different examiners when performing the measurements (as shown in Table 2).

The descriptive statistics results are shown in Table 3, including sample number, gender, and age ranges.

Table 4 shows that a total of 2230 subjects were included in this study: 796 (35.6%) men and 1434 (64.3%) women. The mean age of the participants was 12.2 ± 1.60 years, and there was a statistically non-significant difference between genders.

For Sector 1 of the UMPCs, the total frequency of the right side was 398 (51.02%), including 182 (45.72%) men and 216 (54.27%) women, while left UMPCs were 382 (48.97%), including 172 (45.02%) men and 210 (54.97%) women, respectively.

For Sector 2, the right side was observed in 319 (42.53%) cases, including 118 (36.99%) men and 201 (63%) women, whereas left UMPCs were found in 118 (27.37%) men and 313 (72.62%) women.

For Sector 3 UMPCs, the right side was present in 337 (48.14%), consisting of 133 (39.46%) men and 204 (60.53%) women, while the number of the left side UMPCs was 363 (51.85%), reported in 73 (20.11%) men and 290 (79.88%) women.

No statistically significant association was found between gender and the studied cases if *p* > 0.05.

The training set and a test set are shown in Table 5. Only 20% was used to train the artificial intelligence model, enabling it to learn the relevant radiographic features. The training set has 1787 observations, and the test set has 443 observations. The class distributions were maintained consistently across all sectors to prevent bias.

Table 6 presents information about AI prediction. For Sector 1, the AI reported an accuracy of 99.55%, precision of 100.00%, recall of 97.49%, and F1 of 98.73% for the right-side UMPCs, while for the left side the figures were 99.24%, 100.00%, 95.55%, and 97.72% for accuracy, precision, recall, and F1 value, respectively.

For Sector 2 prediction on the right side, the performance accuracy was 99.73%, precision was 98.15%, recall was perfect at 100.00%, and the F1 score was 99.07%.

For the left-side UMPCs, the prediction value was 98.79% for accuracy, 94.10% for precision, 100.00% for recall, and 96.96% for the F1 value.

Regarding Sector 3 of the UMPCs for the right side, the figures were 99.82% accuracy, 98.83% precision, 100.00% recall, and 99.41% F1. For the left side of UMPCs, the AI achieved an overall accuracy of 99.46%, a precision of 99.72%, a recall of 96.97%, and an F1 equal to 98.32%.

Overall, the DenseNet121 deep learning technique showed consistently high accuracy, exceeding 95% across all metrics, and the right UMPC assessments slightly outperformed the left UMPCs.

**Figure 4:**
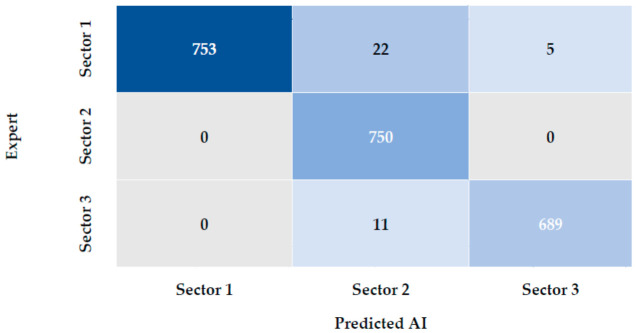
Confusion matrix—severity class based on Cohen’s K: severity class 0.97 and canine side 1.00.

Figure 4 presents the spatial distribution map confusion matrices for all sectors, exhibiting the comparison of the AI and the expert for the sector section. Based on Figure 4, the AI system categorized Sector 1 as Sector 1 or occasionally Sector 2 and, less frequently, as Sector 3. However, the AI system classified Sector 2 as Sector 2 in all images, while predicting Sector 3 as Sector 3, and with less frequency as Sector 2.

In order to compare the distribution of the actual data to the deep learning-predicted data, we used the Mann–Whitney U test. The results indicated no statistically significant difference between the groups (U = 2,502,755 e, *p* = 0.687), which indicates that the observed distributions were largely comparable, as shown in Table 7.

**Figure 5:**
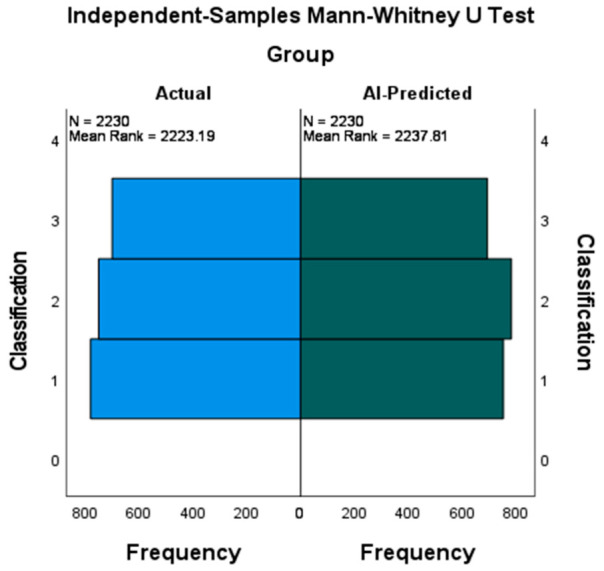
The visualized results of the Mann–Whitney U test as observed in the diagram. There is no statistical significance between the actual and predicted variables.

The medians of both groups were fairly consistent, as shown in the chart in Figure 5 below, with the interquartile ranges overlapping, which indicates little to no significant difference between the two groups’ centers and dispersion, making it impossible to reject the null hypothesis of equal distribution at the α = 0.05 level.

**Figure 6:**
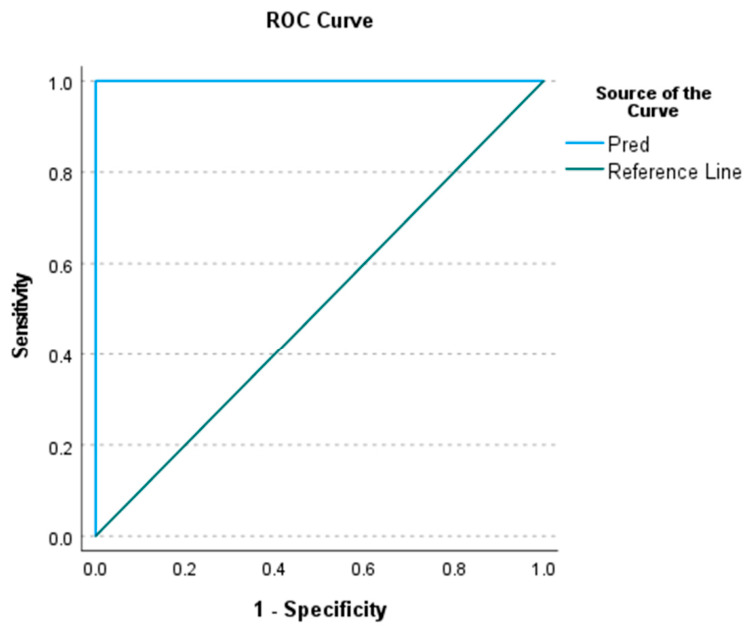
The ROC curve for Sector 1 prediction.

The ROC analysis results for Sector 1 prediction indicate an exceptionally strong classification performance of the model, with an Area Under the Curve (AUC) = 1.000, with a significance level of *p* = 0.000 and a 95% confidence interval of (1.000–1.000). This suggests perfect discrimination between the positive and negative classes (Figure 6).

**Figure 7:**
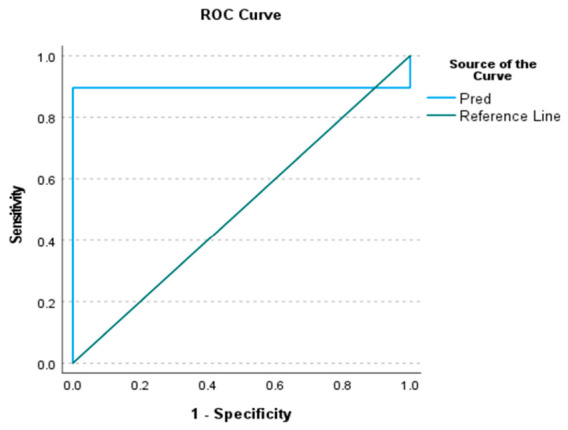
The ROC curve for Sector 3 prediction.

ROC analysis for Sector 2 prediction was identical with no discrepancies, while the ROC analysis for Sector 3 prediction demonstrated a high level of discriminatory power of the model in distinguishing between the two outcome groups. The Area Under the Curve (AUC) was very high (close to 1), indicating that the model performed very well in ranking positive cases higher than negative ones (Figure 7).

## 4. Discussion

This study aims to compare the outcome of a deep learning AI versus orthodontic experts in identifying the position of unerupted maxillary canines in mixed dentition and grouping them according to modified Ericson and Kurol sectors [9].

To ensure the accuracy and consistency of the evaluation process, we randomly selected 106 images from the total of 2230 to ensure the evaluation was conducted consistently and to gauge agreement between examiners (or even the same examiners at different times). The results showed no statistically significant differences (*p* > 0.05), reflecting a strong and reliable level of consistency among the examiners, thus confirming their reliability. This enabled us to confidently use the same standards to look at all 2230 images. Then, the full dataset was used to directly compare the AI’s performance against the human experts.

Traditionally, radiographic assessments of UMPCs have utilized two-dimensional (2D) imaging techniques, such as intraoral periapical, occlusal X-rays, panoramic, and cephalometric radiographs. A panoramic radiograph is the most commonly used imaging technique for the identification of diagnostic parameters aimed at facilitating interceptive treatment planning [26,27], and has the advantage of accessibility, cost-effectiveness, and lower radiation dose compared to CBCT [28].

The clinical implications of this study have the potential to automate orthodontic image analysis and integrate AI into diagnosis and preventive orthodontic treatment planning, facilitating the use of personalized prognostic models. Through training on a large number of images, deep learning algorithms can extract valuable information quickly and automatically, enabling effective disease diagnosis [29].

A subset of the total sample was used in this study to train the artificial intelligence model, enabling it to learn the relevant radiographic features and subsequently analyze the remaining radiographic images in the dataset. After completion of the training phase, the developed model was applied to detect and assess the target findings in all other radiographic images [15,16,17,18,19,20,21,22,23,24,25].

According to this study, a deep learning-based system for automatic landmark identification and parameter generation significantly improves accuracy and precision by more than 98% (Table 6), as well as efficiency by reducing manual effort. This higher prediction was especially prominent in the grouping of UMPCs for Sectors 1, 2, and 3. The AI system in our study follows a five-phase image processing approach (Figure 3). At first, it inputs the images (panoramic radiographs), then performs pre-processing (pixel rescaling, image resizing, augmentation, resolution, and enhancement). AI detection (AI automatically identifies landmarks) then generates data (segmentation and classification), and finally predicts the outcome (predictive formula). In addition, the AI performance can be improved by a multiphase technique [30]. However, with this multiphase approach, some issues may occur. For instance, the accuracy of the final stages is related to the accuracy of the earlier phases. This initial image distortion, if present, can disrupt later phases [31]. For that reason, we excluded images with artifacts, defects, or distortion.

Deep learning demonstrated the best performance with the highest sensitivity (Sector 1: right 99.55%, left 99.24% for accuracy, with 100% precision for both sides). For Sector 2, the right side predicted 99.73%, and the left had 98.79% accuracy, with 98.15% and 94.10% precision for right and left. Sector 3 showed an accuracy value of 99.82% for the right side and 99.46% accuracy for the left side, with a precision of 98.83% and 99.72% for the right and left sides; these findings were consistent with the findings of Bardideh et al. [30].

Several studies in the medical field have shown that pretrained models often produce higher precision with less data compared with training from less distorted images and allow clinicians to understand the rationale behind predictions and make informed treatment decisions [32,33,34].

Based on our results, the AI model’s accuracy and precision in categorizing UMPCs were comparable to those of the expert, who performed classification only by examining the panoramic radiographs. The AI produced comparable results in categorizing UMPCs in relation to incisors. A previous study by Zhang C et al. [35] concluded an AI accuracy of 95% in diagnosing UMPCs using panoramic radiographs. However, different research by Dodge et al. found that images that are not distorted would be detected better by AI systems [36].

While this article does not explicitly state that AI is definitively replacing human experts in diagnosing UMPCs, the high accuracy and reliability of AI models, combined with manual refinement, suggest that AI has the capability to match human accuracy in this specific diagnostic task.

It appears that artificial intelligence (AI) will play a significant role in the future of orthodontics, with the promise of providing more accurate and efficient treatment plans. For orthodontic professionals to fully utilize AI, ongoing education is essential. The future of orthodontics and artificial intelligence (AI) together has extensive potential to improve treatment outcomes and enhance the interceptive orthodontic care environment [37].

The results of our study retained the null hypothesis and found a statistically non-significant difference between the two groups (*p* < 0.05); see Table 7.

Finally, the median values between the two groups were relatively close, as displayed in the graph below (Figure 5). The Interquartile Ranges (IQRs) for each group overlapped, demonstrating little to no difference between the centers and the spread of the two groups. As can be seen in the charts, there is only a slight difference between the groups. However, this difference is not significant, and therefore it could be due to chance (e.g., sampling error) and may not actually show a difference between the groups. Therefore, we can conclude that there is no real difference between the deep learning model and an expert when it comes to measuring that certain variable. Because of this, we cannot reject the null hypothesis that the distributions are equal at an α = 0.05 significance level.

To confirm this perfect prediction, Sector 1 deep learning prediction was also drawn for the ROC analysis in Figure 6, giving an AUC of 1.000 and optimal results in all measures of evaluation (Gini Index, K-S statistic, sensitivity, and precision). Further, the Kolmogorov–Smirnov (K-S) statistic equaled 1 at a cut-off value of 1.5, which means maximum separation between the cumulative distribution of the two classes; in other words, a Gini Index of 1 further confirms this perfect predictive power, as the Gini Index is directly related to AUC (Gini = 2 × AUC − 1). These outcomes reflect a best-case scenario of a predictive system, but care must be taken when interpreting these results. The ideal outcome of ‘perfect’ is rare in practice and may represent problems like overfitting or lack of generalizability in the predictive system.

This is also confirmed by the precision and recall results: The precision values are extremely high (the majority of them are ≥0.96). The number of missed positive cases is zero (recall or sensitivity is 1.0).

The ROC analysis shows that the model for predicting Sector 3 has a high AUC and statistically significant results (Figure 7), suggesting that the model has good predictive ability. The chosen cut-off point provides a practical compromise between sensitivity and specificity, thus telling us that deep learning is applicable in the real world. The best cutoff (based on ROC coordinates) is a compromise between sensitivity and specificity; our findings are in agreement with those reported by de Araujo et al. [38]. The precision–recall relationship is another indicator of the effectiveness of the model, as the model is correct almost all the time when predicting a positive case. This is especially crucial when the data set is not balanced.

In summary, the model exhibited highly accurate and consistent classification capabilities, showing promise for real-world use (subject to further validation) [39]. The deep learning system tested in this study was able to detect an unerupted upper canine position in relation to the incisors using a panoramic radiograph alone. The applications of such a system may extend to algorithmic preventive orthodontic diagnosis, patient record analysis, archiving, and follow-up evaluations. Furthermore, comparing our results with those of manual examinations by expert clinicians, the AI system can provide faster outcomes, improved accessibility, and higher levels of precision and accuracy.

Future studies should include larger, multicenter datasets with a more diverse population to enhance the generalizability of the proposed deep learning model. Moreover, the model should be externally validated on an independent dataset to assess the robustness, reliability, and clinical validity of the model to other patients and health care settings. The final recommendation is to assess the performance of the proposed deep learning model with respect to cone-beam computed tomography (CBCT) as a three-dimensional imaging.

## 5. Conclusions

This study finds that the deep learning-based system tested significantly reduced the time and human resources required for landmark identification and parameter generation, making the diagnostic process more efficient in interceptive orthodontics. However, the existing model exhibits certain limitations, and unresolved challenges remain. Future research should aim to develop a more dependable and precise model for predicting unerupted canines by leveraging AI.

Our AI model was optimal in predicting the eruption of permanent maxillary canines as an interceptive measure using panoramic radiographs, showing high accuracy, precision, and recall. Furthermore, the system was proven to be comparable in predicting the eruption of permanent maxillary canines compared to an expert orthodontist.

## Figures and Tables

**Table 1:** Inter-examiner calculation.

Group	Examiners	*N*	Mean Rank	Sum of Ranks	Mann–Whitney U	*p*-Value	Decision
Test	Inter	106	104.04	11,028.00	5357	0.614	NS
	Intra	105	107.98	11,338.00			
	Total	211					

**Table 2:** Inter-examiner calculation.

Group	Examiners	*N*	Mean Rank	Kruskal–Wallis H	*p*-Value	Decision
Test	Intra1	106	156.63	0.394	0.821	NS
	Inter1	106	158.20			
	Inter2	106	163.67			
	Total	318				

**Table 3:** Descriptive statistics.

Parameters	No. (%)	Mean Ages (SD)	Min. Age	Max. Age	Range
Gender					
Male	796 (35.7%)	12.12 (1.61)	8.9	14.7	5.8
Female	1434 (64.3%)	12.32 (1.59)	8.9	15.0	6.1
Total (subjects)	12.25 (1.60)	12.25 (1.60)	8.9	15.0	6.1
Un-erupted canine side					
Right	1054 (47.3%)	12.12 (1.61)	8.9	14.7	5.8
Left	1176 (52.7%)	12.32 (1.59)	8.9	15.0	6.1
Total (subjects)	2230 (100%)				

**Table 4:** Distribution of Sectors 1, 2, and 3 by gender.

Gender										Chi-Square *p*-Value	Decision
				Male		Female		Total			
				No.	%	No.	%	No.	%		
Sectors	Sector 1	Side	Left	172	45.0%	210	55.0%	382	48.97%	0.039	S ^1^
			Right	182	45.7%	216	54.3%	398	51.03%		
	Sub-Total			354		426		780			
	Sector 2	Side	Left	118	27.4%	313	72.6%	431	57.47%	0.005	HS ^2^
			Right	118	37.0%	201	63.0%	319	42.53%		
	Sub-Total			236		514		750			
	Sector 3	Side	Left	73	20.1%	290	79.9%	363	51.86%	0.000	VHS ^3^
			Right	133	39.5%	204	60.5%	337	48.14%		
	Sub-Total			206		494		700			
Grand Total				796		1434		2230			

^1^: S = Significant. ^2^: HS = Highly significant. ^3^: VHS = Very high significance.

**Table 5:** The training set and the test set.

UMPCs		Test	Train
		NO. 20 (%)	NO. 80 (%)
Sector1	Right	79 (19.8%)	319 (80.2%)
	Left	76 (19.9%)	306 (80.1%)
	Total	155 (19.9%)	625 (80.1%)
Sector 2	Right	63 (19.7%)	256 (80.3%)
	Left	86 (20.0%)	345 (80.0%)
	Total	149 (19.9%)	601 (80.1%)
Sector 3	Right	67 (19.9%)	270 (80.1%)
	Left	72 (19.8%)	291 (80.2%)
	Total	139 (19.9%)	561 (80.1%)
Total		443	1787

**Table 6:** AI’s accuracy, precision, recall, and F1 score outcomes.

UMPCs		Side	
		Right%	Left%
Sector 1	Accuracy	99.55%	99.24%
	Precision	100.00%	100.00%
	Recall	97.49%	95.55%
	F1 Score	98.73%	97.72%
Sector 2	Accuracy	99.73%	98.79%
	Precision	98.15%	94.10%
	Recall	100.00%	100.00%
	F1 Score	99.07%	96.96%
Sector 3	Accuracy	99.82%	99.46%
	Precision	98.83%	99.72%
	Recall	100.00%	96.97%
	F1 Score	99.41%	98.32%

**Table 7:** Hypothesis test summary.

Null Hypothesis	Test	Sig. ^a,b^	Decision
The distribution of sectors is the same across categories of sectors.	Independent-Samples Mann–Whitney U Test.	0.687	Retain the null hypothesis.

^a^ The significance level is 0.050. ^b^ Asymptotic significance is displayed.

## Data Availability

The data presented in this study are available on request from the corresponding author due to privacy and ethical concerns; the data have been retrieved from archived records of the Oral Diagnosis Department, College of Dentistry, Hawler Medical University. These records can only be accessed with permission from the Oral Diagnosis Department within the institution’s rules and policies.

## Abbreviations

The following abbreviations are used in this manuscript:

AI	Artificial intelligence
ML	Machine learning
DL	Deep learning
CNNs	Convolutional neural networks
UMPCs	Unerupted maxillary permanent canines
